# GeO_2_ Nanoparticles Decorated in Amorphous Carbon Nanofiber Framework as Highly Reversible Lithium Storage Anode

**DOI:** 10.3390/molecules28186730

**Published:** 2023-09-21

**Authors:** Wenhe Xie, Congcong Liu, Chen Hu, Yuanxiao Ma, Xuefeng Li, Qian Wang, Zhe An, Shenghong Liu, Haibin Sun, Xiaolei Sun

**Affiliations:** 1 Key Laboratory of Microelectronics and Energy of Henan Province, Xinyang Normal University, Xinyang 464000, China; liucc528@163.com (C.L.); yczm100520@163.com (C.H.); 18129894906@163.com (Y.M.); lxuefeng0827@163.com (X.L.); 17627269682@163.com (Q.W.); 13353729786@163.com (Z.A.); liush@xynu.edu.cn (S.L.); sunhaibin@xynu.edu.cn (H.S.); 2 School of Materials Science and Engineering, Smart Sensing Interdisciplinary Science Center, Tianjin Key Lab for Rare Earth Materials and Applications, Center for Rare Earth and Inorganic Functional Materials, Nankai University, Tianjin 300350, China

**Keywords:** GeO_2_, amorphous, lithium-ion batteries, alloy reaction, conversion reaction, reversible electrode

## Abstract

Germanium oxide (GeO_2_) is a high theoretical capacity electrode material due to its alloying and conversion reaction. However, the actual cycling capacity is rather poor on account of suffering low electron/ion conductivity, enormous volume change and agglomeration in the repeated lithiation/delithiation process, which renders quite a low reversible electrochemical lithium storage reaction. In this work, highly amorphous GeO_2_ particles are uniformly distributed in the carbon nanofiber framework, and the amorphous carbon nanofiber not only improves the conduction and buffers the volume changes but also prevents active material agglomeration. As a result, the present GeO_2_ and carbon composite electrode exhibits highly reversible alloying and conversion processes during the whole cycling process. The two reversible electrochemical reactions are verified by differential capacity curves and cyclic voltammetry measurements during the whole cycling process. The corresponding reversible capacity is 747 mAh g^−1^ after 300 cycles at a current density of 0.3 A g^−1^. The related reversible capacities are 933, 672, 487 and 302 mAh g^−1^ at current densities of 0.2, 0.4, 0.8 and 1.6 A g^−1^, respectively. The simple strategy for the design of amorphous GeO_2_/carbon composites enables potential application for high-performance LIBs.

## 1. Introduction

Billions of lithium-ion batteries (LIBs) occupy the booming electrochemical energy storage market due to their high output voltage, high energy density, long cycling life and so on [1,2,3]. The performance of LIBs is mainly determined by the applied anode and cathode materials. When LIBs are charged, lithium ions come out of the lattice of the cathode material and are inserted into the anode material after passing through the electrolyte. During the discharge process, lithium ions are extracted from the anode electrode material and returned to the lattice of the positive electrode material after passing through the electrolyte. In this way, the potential difference between the anode and cathode materials is the working voltage when lithium ions are inserted and removed from the two electrodes. There are four main types of cathode materials for LIBs: lithium cobaltate, lithium manganate, ferrous lithium phosphate and ternary. The evolution of electrode materials will bring enormous commercial and economic value benefits. One promising research point is focused on updating the current graphite anode, whose low theoretical capacity (372 mAh g^−1^) and poor rate performance cannot fully meet the growing demand; therefore, researchers have devoted numerous efforts to the development of novel advanced anode materials [4,5,6,7,8].

From the periodic table of elements, we can conclude various kinds of lithium storage materials. Briefly, many transition metal elements are regarded as inactive, while their oxides, such as Fe_2_O_3_, MnO, NiO and CoO [9,10,11,12,13], are active and can reversibly react with lithium at a relatively high potential (the corresponding conversion reaction equation is MO + Li ↔ M + Li_2_O, where M represents transition metal). In addition, some group 3 elements (Ga, In) [14,15], group 4 elements (Si, Ge, Sn) [16,17,18] and group 5 elements (Sb, Bi) [19,20,21] can form alloys with lithium at relatively low potentials (the corresponding alloying equation is N + Li ↔ NLi, where N presents transition group 3, 4 and 5 elements) [22,23]. However, the lithium storage mechanism of group 3–5 element-based oxides is rather complicated, and the related electrochemical reaction consists of alloying and conversion reaction processes [24,25,26,27]. It is worth noting that the latter suffers insufficient kinetics with lithium; thus, the corresponding conversion reaction displays a low degree of reversibility in many cases.

Germanium oxide (GeO_2_) has attracted increasing attention in view of its low working platform, high chemical stability and high theoretical lithium storage capacity [28,29,30,31]. One GeO_2_ molecule can store 8.4 Li atoms considering the fully reversible alloying and conversion stages, and the delivered capacity reaches up to 2152 mAh g^−1^. However, regardless of these fascinating potentials, the actual lithium storage performance of GeO_2_ electrodes is seriously hindered by the dramatic volume change that occurs during the lithiation/delithiation process. The repeated volume expansion/contraction destroys the electrode, and as a result, the active material is stripped from the current collector. To make matters worse, the volume expansion also cracks the solid electrolyte interphase (SEI) layer, which originates from the decomposition of the electrolyte on the interface between the electrolyte and active material, inducing repeated growth of the SEI layer and excessive consumption of the electrolyte. In addition, GeO_2_ also suffers inherent low electron/ion conductivity. These disadvantages render sluggish electrochemical reactions [32,33]; thus, many GeO_2_ electrodes deliver quite low Coulombic efficiency (especially for the initial cycle), low reversible capacity and poor cycling and rate performance.

Various strategies have been developed to respond to the above shortcomings and improve the lithium storage performance of GeO_2_ electrodes. Primarily, GeO_2_ samples are operated at the nanoscale level (nanosheets [34], nanoparticles [35], nanotubes [36], nanofibers [37], etc.) to enhance the reactivity of the electrode. These nanomaterials supply convenient lithium ion diffusion paths and abundant physical spaces for volume changes, which accelerates the electrochemical reaction process and expands the reversible capacity. Second, introducing a conductive matrix, such as carbon [38,39,40] or metal [41,42,43], with GeO_2_ enhances the electronic conductivity, which relieves electrode polarization and upgrades the rate performance to a certain degree [44,45]. However, GeO_2_ nanomaterials tend to agglomerate in repeated lithium intercalation/deintercalation reactions; thus, the initial morphology and structure may be gradually destroyed. As a consequence, the electrochemical reversibility worsens (especially for the conversion reaction stage) in the cycling process. Thus, it is quite meaningful to respond to the electrode conductivity, volume expansion and agglomeration to promote superior GeO_2_ anodes with durable alloying and conversion reactions in repeated electrochemical processes.

In this work, we disperse GeO_2_ particles into carbon nanofibers via facile electrospinning and thermal treatment technology. The uniformly distributed GeO_2_ nanoparticles are embedded in the carbon nanofiber framework, and the high specific area supplies abundant electrochemical reaction sites for fast lithium diffusion. Moreover, the carbon framework not only buffers the induced volume changes but also prevents the active material from agglomerating. The composite nanofiber maintains structural stability in the repeated lithiation/delithiation process. In addition to the typical ion diffusion controlled reaction, the prepared GeO_2_ carbon composite nanofiber electrode also delivers capacitive-controlled capacities of 21.3%, 26.5%, 30.8%, 34.5% and 37.4% at scanning speeds of 0.2, 0.4, 0.6, 0.8 and 1.0 mV s^−1^, respectively. The corresponding electrodes exhibit highly reversible lithium storage reactions (including alloying and conversion processes) during the long-term charging and discharging process.

## 2. Results and Discussion

**Figure 1:**
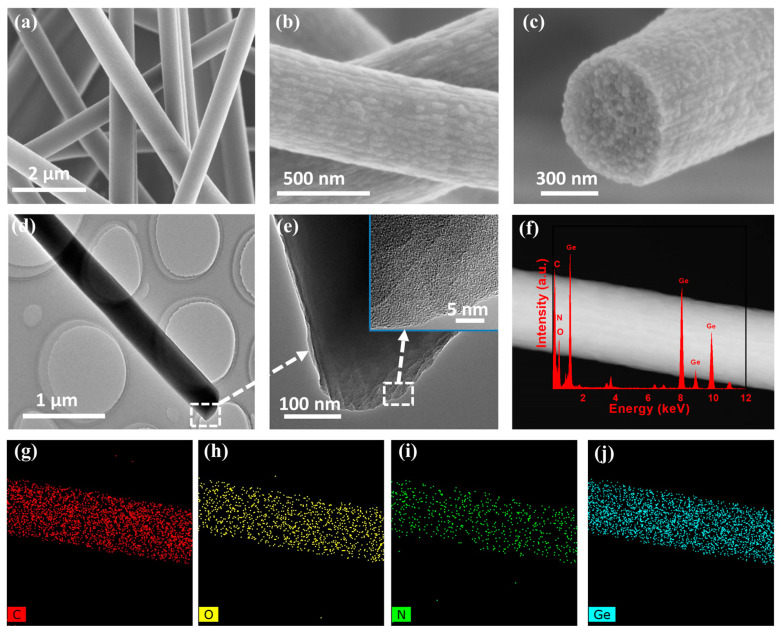
Morphological characterization of the A-GeO_2_-CNFs sample. (**a**–**c**) SEM images; (**d**,**e**) TEM images; (**f**) STEM image and EDX spectrum; (**g**–**j**) elemental mapping.

The low-dimensional morphology of the A-GeO_2_-CNFs sample was characterized by scanning electron microscopy (SEM) and transmission electron microscopy (TEM), as shown in Figure 1. From the low-magnification SEM image (Figure 1a), we can see that numerous electrospun nanofibers randomly overlap with a diameter distribution ranging from 200–500 nm. Moreover, the enlarged SEM images in Figure 1b,c demonstrate that the nanofibers have relatively rough surfaces, which form due to solvent volatilization and film rupture during the carbonization process. Such a rough structure enhances the lithium ion diffusion interface and benefits from convenient electrochemical reactions. The TEM image in Figure 1d records a single nanofiber with a dark homogeneous distribution. No nanoparticles can be found from the enlarged edge of the nanofiber in Figure 1e, and no obvious lattice stripe can be observed from the high resolution TEM image (the inset in Figure 1e), indicating that both the carbon and GeO_2_ active materials are amorphous structures. In addition, the STEM (scanning transmission electron microscopy) image in Figure 1f shows the homogeneous distribution of Ge in the composite nanofiber sample. The EDX (energy dispersive X-ray) spectroscopy in Figure 1f (red curve) displays the existence of C, O, N and Ge in the present nanofiber sample, and the related elemental mapping (Figure 1g–j) proves that these four elements are evenly mixed and distributed in the nanofiber, while the Ge element is rooted in the oxidation of the germanium source.

**Figure 2:**
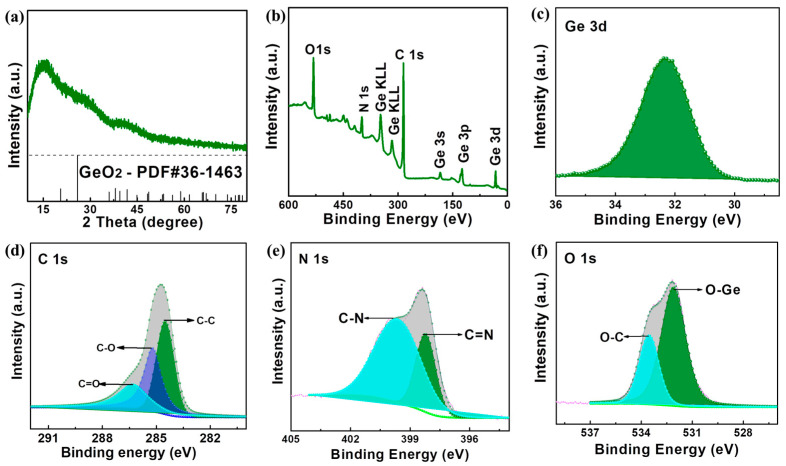
Composition characterization of the A-GeO_2_-CNFs sample. (**a**) XRD pattern. (**b**) Full XPS spectrum. (**c**) High-resolution Ge 3d peak. (**d**) High resolution of the C 1s peak. (**e**) High resolution of the N 1s peak. (**f**) High resolution of the O 1s peak.

Next, the composition characterization of the present A-GeO_2_-CNFs sample is shown in Figure 2. No obvious diffraction peaks can be observed from the XRD pattern in Figure 2a, proving that both carbon and GeO_2_ are amorphous structures, which is consistent with the above TEM characterization. Furthermore, to accurately investigate the sample element types and valence states, XPS measurements were carried out to acquire the detailed chemical bonding energy. From the full XPS spectrum in Figure 2b, we can clearly find the Ge, O, C and N elements, which is consistent with the TEM characterization. The high resolution of the Ge 3d peak (Figure 2c) at approximately 32.5 eV corresponds to the Ge-O bond in the sample, and no obvious low valent germanium (Ge^2+^, or Ge) peaks can be fitted, implying the formation of GeO_2_ in the carbon nanofiber. The precise C 1s peak in Figure 2d can be fitted into three peaks with bonding energies of 284.5, 285.3 and 286.7 eV, which correspond to C-C, C-O and C-N groups, respectively. In addition, the precise N 1s peak (Figure 2f) consists of pyridinic N (at approximately 400.2 eV) and pyrrolic N (at about 398.7 eV) [46,47,48]. The C and N elements originate from the carbonized PVP and PAN binders. Furthermore, the precise O 1 s peak (Figure 2f) can be divided into an O-C group at 533.6 eV and an O-Ge group at 532.1 eV, proving that a small amount of O is derived from the carbon source and that the other large amount of O originates from the oxidation of the germanium source. Based on the above results, we can conclude that the present carbon nanofibers are doped in situ with abundant N and O elements. Studies have shown that this doping can improve electrode conductivity, lower the ion diffusion barrier and increase the number of lithium storage sites [49,50,51].

**Figure 3:**
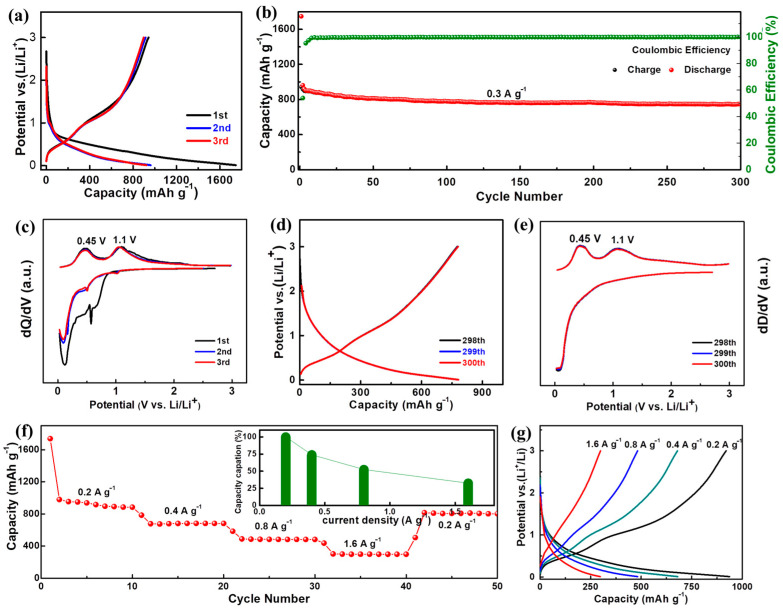
Electrochemical characterization of the electrode composed of A-GeO_2_-CNFs. (**a**,**c**) Charge–discharge curves and differential capacity curves of the initial three cycles. (**b**) The constant current cycle test at 0.2 A g^−1^. (**d**,**e**) Charge–discharge curves and differential capacity curves of the 298–300th cycles. (**f**) Rate performance test. (**g**) Charge–discharge curves at various current densities.

Subsequently, a series of electrochemical characterizations were performed to evaluate the lithium storage performance of the A-GeO_2_-CNFs based electrodes. Figure 3a presents the representative charge–discharge curves of the initial three cycles in the voltage window range from 0.01–3.0 V. One can see that the first discharge capacity (1746 mAh g^−1^) is much higher than the corresponding charge capacity (942 mAh g^−1^), and the relatively large irreversible capacity (804 mAh g^−1^) and low initial Coulombic efficiency (54%) can be mainly attributed to the irreversible decomposition of the electrolyte into the SEI layer and some inevitable side reactions. The electrode composed of A-GeO_2_-CNFs demonstrates highly overlapped curves from the second cycle, indicating that the formed SEI layer is quite stable and beneficial for convenient lithium diffusion. The detailed constant current cycle test at 0.3 A g^−1^ is recorded in Figure 3b. From the red dot diagram, we can see that the capacity decays very slowly during the whole cycling process, and the corresponding reversible capacity is 747 mAh g^−1^ after 300 cycles at a current density of 0.3 A g^−1^, which is approximately 79.3% of the initial reversible capacity value. In addition, the Coulombic efficiency reaches more than 97% after three cycles and nearly 99% after five cycles, implying a highly reversible discharge–charge reaction process in the subsequent test.

To better investigate the lithium storage mechanism, we plot the differential capacity curve of the electrode composed of A-GeO_2_-CNFs for the initial three cycles in Figure 3c. From the differential first discharge capacity curve, we can see a unique broad peak range from 0.4–1.0 V, which involves the growth of the SEI layer. Notably, two obvious peaks at 0.45 and 1.1 V can be clearly observed in the differential charge capacity curves, which correspond to the dealloying (formation of Ge) and further delithiation reaction (formation of GeO_2_), respectively. Notable, no obvious reduction peaks can be recorded from the differential discharge capacity curves, proving that GeO_2_ and carbon are amorphous structures, which is in good agreement with the above XRD and TEM measurements. Figure 3d,e show the charge–discharge curves and differential capacity curves of the 298–300th cycles, respectively, from which one can see that the delivered charge–discharge curves and redox peaks are consistent with those of the 2nd–3rd cycles in Figure 3a,c. Such characteristic peaks twice reflect that the dealloying (Ge + 4.4Li ↔ GeLi_4.4_) and conversion reaction (GeO_2_ + 4Li ↔ Ge + 2Li_2_O) is highly reversible during the whole cycling process. In addition, we can see that the curves of the 298–300th cycles are almost the same, indicating that the electrode has a highly stable and reversible charge and discharge ability.

Figure 3f presents the rate performance of the electrode composed of A-GeO_2_-CNFs. The delivered reversible capacities are 933, 672, 487 and 302 mAh g^−1^ at current densities of 0.2, 0.4, 0.8 and 1.6 A g^−1^, respectively. The corresponding capacity retention rates are 100%, 72.0%, 52.2% and 32.4%, as shown in the inset. It is worth noting that when the current is adjusted back to 0.2 A g^−1^, the electrode capacity is restored to 807 mAh g^−1^, which is approximately 86.4% of the initial value. The typical charge–discharge curves at different current densities are recorded in Figure 3g. From the charge profiles, we can see that the two oxidation plateaus weakened under a relatively high current density, and this phenomenon can be attributed to the polarization of the electrode.

**Figure 4:**
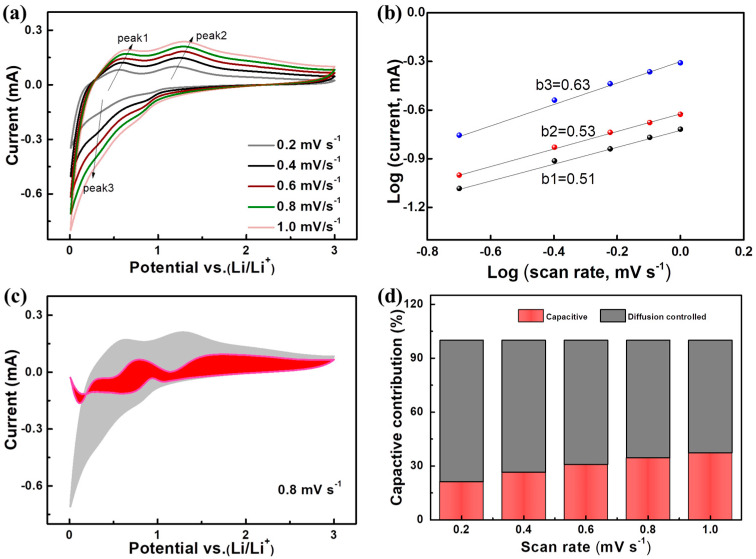
(**a**) CV curves of the electrode composed of A-GeO_2_-CNFs at unequal scan rates from 0.2 to 1.0 mV s^−1^; (**b**) log (i)/log (v) plots; (**c**) typical contribution of diffusion to the overall charge storage at 0.8 mV s^−1^; (**d**) contribution ratio at different scan rates.

The superior lithium storage performance of the A-GeO_2_-CNFs sample is rooted in its convenient electrochemical reaction. To better study the Li storage mechanism, we measured the cyclic voltammetry (CV) at different rates ranging from 0.2–1.0 mV s^−1,^ as shown in Figure 4a. Notably, two obvious oxidation peaks can be recorded during the whole anodic scanning process, indicating that both the alloying and conversion reactions are highly reversible even at high scanning speeds. The induced current contribution can be evaluated from the variable-speed scanning CV via the following equation:

i = a*v*^b^,

(i) = log (a) + blog (*v*)

**Figure 5:**
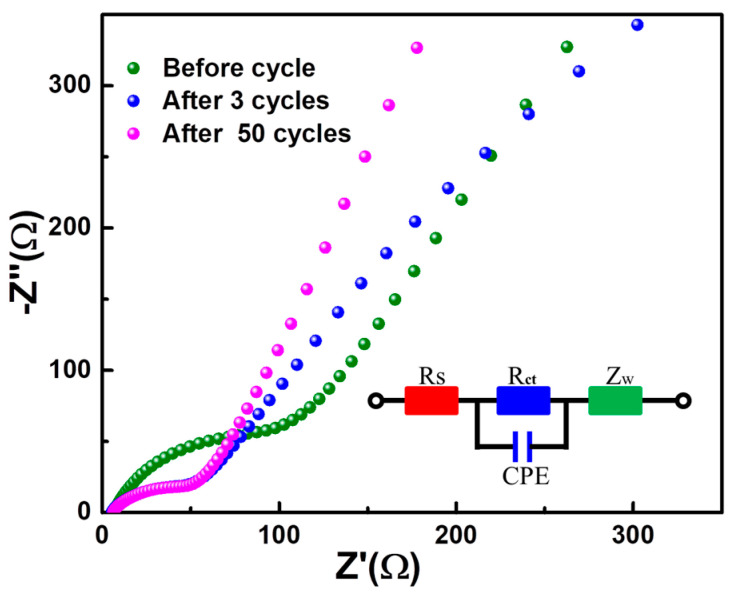
Nyquist plot of the as-prepared electrode composed of A-GeO_2_-CNFs and the equivalent circuit diagram.

*v* and i represent the scan speed and peak current, respectively. a and b are variable parameters. The value of b can be obtained by calculating the slope. When b = 0.5, the lithium storage mechanism originates from ion diffusion-controlled behavior, while b = 1 implies that the lithium storage behavior is dominated by a capacitive surface adsorption process. Figure 4b shows that the calculated b values of peak 1, peak 2 and peak 3 are 0.51, 0.53 and 0.63, respectively. The value is closer to 0.5 and less than 1, indicating that the total Li storage capacity mechanism consists of a large proportion of diffusion-controlled behavior and a small proportion of capacitive behavior. The typical capacitive contribution distribution vs. total capacity at a scanning speed of 0.8 mV s^−1^ is demonstrated in Figure 4c. In addition, the detailed percentages of diffusion-controlled and capacitive-controlled values are depicted in Figure 4d. The capacitive-controlled contributions are 21.3%, 26.5%, 30.8%, 34.5% and 37.4% at scanning speeds of 0.2, 0.4, 0.6, 0.8 and 1.0 mV s^−1^, respectively. The increased percentage value implies that the capacitance behavior is more resistant to polarization compared with ion diffusion-controlled behavior at a high current density. In addition, electrochemical impedance spectroscopy (EIS) was applied to investigate the cell kinetics, as shown in Figure 5. From the Nyquist data, we can see that dense dots form a semicircle at the high-medium frequency region, and relatively sparse dots form a line at the low frequency region. The equivalent circuit diagram consists of R_s_ (solution resistance) and R_ct_ (charge transfer), CPE (constant phase element) and Z_w_ (Warburg diffusion impedance). The charge transfer resistance is the core index of the cell, and from the fitting value, we can see that the R_ct_ values before cycle, after three cycles and after 50 cycles are 124.6, 62.5 and 64.2 Ω, respectively, indicating that the resistance of the A-GeO_2_-CNFs electrode is relatively small and stable in the cycling process.

**Figure 6:**
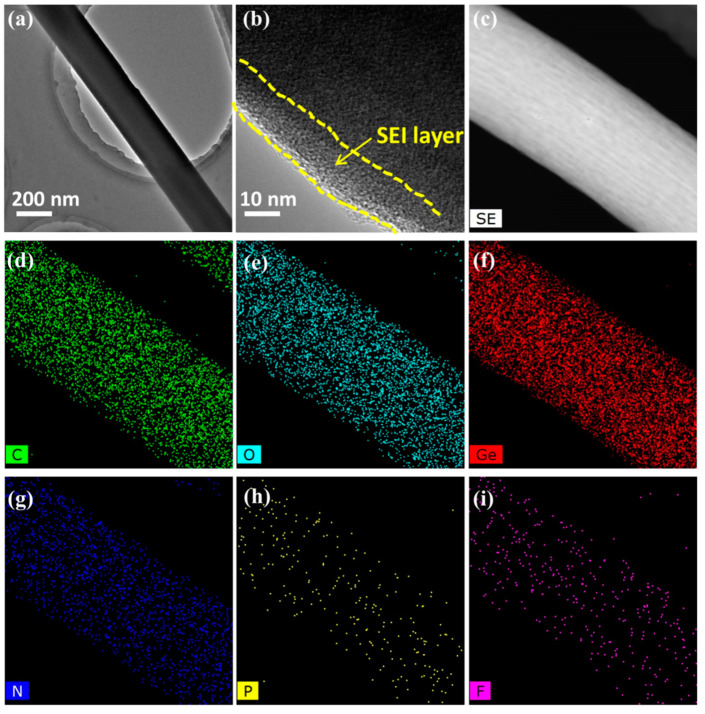
Morphological characterization of the cycled A-GeO_2_-CNFs sample. (**a**,**b**) TEM images; (**c**) STEM image; (**d**–**i**) elemental mapping.

To further investigate the superior lithium storage mechanism of the present electrode composed of A-GeO_2_-CNFs, we disassembled the cycled cell and removed the working electrode in a glove box. After washing with PC (propylene carbonate) solvent, the sample was characterized by TEM, as shown in Figure 6. From the low-magnification image (Figure 6a), we can see that the sample still maintains a large-scale nanofiber structure, which is similar to the pristine sample in Figure 1d. In addition, the enlarged TEM image (Figure 6b) shows the formed SEI layer with a thin thickness of approximately 10 nm. Moreover, the secondary characterization STEM and mapping are displayed in Figure 6c–i, which clearly demonstrate the existence of C, O, Ge, N, P and F elements. Compared with pristine nanofibers, the additional weak N and F elemental signals of the cycled nanofiber sample come from the decomposition of a small amount of LiPF_6_ electrolyte. These measurements indicate excellent structural stability of the nanofiber in the repeated charge–discharge process.

## 3. Materials and Methods

### 3.1. Synthesis of Sample

The composite nanofibers sample involves gel solution preparation, electrospinning and thermal annealing. The detailed synthesis steps can be summarized as follows: First, 0.8 g PAN (polyacrylonitrile, purchased from Sigma Aldrich, the average molecular weight is approximately 150,000) and 1.2 g PVP (polyvinylpyrrolidone, purchased from Sigma Aldrich, the average molecular weight is about 1,300,000) binders as carbon sources were added to 14 g of DMF (N,N-dimethylformamide, purchased from Innochem) solvent, and the mixed solution was kept in an oven at 80 °C for about 2 h to dissolve the binders. Then, 0.9 g of germanium tetrachloride was added and stirred to obtain a homogeneous gel solution.

Next, the prepared gel was injected into a 10 mL disposable syringe with a stainless steel needle with a diameter of 0.5 mm, and the syringe was placed on an electrostatic spinning machine (WL-2, Beijing Aibo Zhiye Ion Technology Limited Company, Beijing, China). The applied positive voltage and negative high voltages were 15 and −1.5 kV, respectively. Notably, the positive high voltage wire and negative high voltage wires were connected with the stainless steel needle and grounded roller, respectively. The electrostatic spinning product was collected by the roller with a rotation speed of 50 revolutions per minute, and the roller was covered with aluminum foil. The electrospinning distance from the needle tip to the roller was approximately 15 cm. The set electrospinning colloid solution output speed was about 1.0 mL h^−1^. The whole electrospinning operation was carried out at room temperature and in an open environment.

Subsequently, the obtained spinning product with aluminum foil was dried in an oven (at 80 °C for approximately 2 h) to evaporate the excess solvent. Then, the product was stripped from the aluminum foil and placed in a porcelain boat. After that, the solidified fiber product was preoxidized in air at 280 °C for approximately 4 h. Finally, the amorphous GeO_2_ and carbon nanofibers (A-GeO_2_-CNFs) sample was obtained after further carbonization in an argon atmosphere at 550 °C for another 4 h.

### 3.2. Materials Characterization

The crystalline microstructures of the samples were examined by XRD (X-ray diffraction, Rigaku D/Max-2400 diffractometer, Cu Kα radiation, λ = 0.15406 nm) in the range of 10–80° with a scanning speed of 2° min^−1^. The applied tube voltage and current were 40 kV and 30 mA, respectively. The element types, valence states and detailed chemical bonding energy were investigated by X-ray photoelectron spectroscopy (XPS, Kratos AXIS Ultra DLD, Al Kα probe beam, the applied photoelectron energy h*v* = 1486.6 eV), and the nanofiber sample was pressed into a film by a tablet press and evacuated for 1 day before testing. The fitting software of the high-resolution spectra was XPS Peak.

To prepare the FE-SEM (field-emission scanning electron microscopy, S-4800, Hitachi) characterization, the sample is glued to the metal table with conductive glue. In addition, a TEM (transmission electron microscopy, FEI, Tecnai G^2^ F20) with EDX (energy dispersive X-ray) spectroscope was applied to acquire the detailed microstructure and composition. During the sample preparation, the sample was first put into an alcohol solution and ultrasonicated for about 10 min. Next, a dropper was used to draw a small amount of supernatant onto the micro-grid. Finally, the micro-grid with the sample was dried for a few minutes to evaporate alcohol.

### 3.3. Synthesis of Cells

The lithium storage performance of the as-prepared A-GeO_2_-CNFs sample was tested by assembling half cells. The A-GeO_2_-CNFs sample was prepared into a working electrode, and lithium foils were used as a counter electrode and a reference electrode. The electrodes composed of A-GeO_2_-CNFs were prepared using a typical coating procedure. Briefly, the active material sample, acetylene black conductive agent and PVDF (polyvinylidene fluoride) binder were mixed at a mass ratio of 80:10:10. These mixtures were put into a mortar. After vigorous grinding for approximately 2 h, NMP (N-Methyl pyrrolidone) solvent was added to form a viscous slurry, which was coated on copper foil and dried in a vacuum oven at 120 °C for 10 h. Subsequently, the copper foil was cut into 12 mm discs as the working electrode. The active material loading for each electrode was about 0.8–2.0 mg. Next, the working electrodes, Whatman GF/F-90 glass fibers separators, lithium metal foils (purchased from Tianjin Zhongneng Lithium Industry Co., Ltd. Tianjin, China), electrolyte (1 M LiPF_6_ dissolved in ethylene carbonate (EC) and dimethyl carbonate (DEC) with a volume ratio of 1:1, purchased from DoDoChem, Suzhou, China), stainless steel gasket, spring leaf, CR 2032 positive and negative battery shells were placed in a high purity argon gas-filled glove box (Etelux, Beijing, China, O_2_ < 1 ppm, H_2_O < 1 ppm). To assemble the half cells, lithium foil was placed in the negative battery shell, and the glass fibers separator and working electrode were carefully placed on the surface of the lithium foil in turn. Next, 100 μL of electrolyte was dripped into the negative battery shell to ensure sufficient infiltration by a pipette gun. Subsequently, a stainless steel gasket and spring leaf were placed in the middle position in turn, and the positive battery shell was placed at the top of the structure. The positive and negative battery shells were clamped into the mold of the pelletizer by an insulating tweezer, and half cells were sealed under pressure of about 5–7 MPa. It is particularly noteworthy that we avoided direct contact between the positive and negative electrodes during the operation. The open circuit voltage of these assembled half cells is generally higher than 2.5 V. After aging for approximately 12 h, the prepared cells were evaluated by an electrochemical workstation (CHI-660E, Chenhua, Shanghai, China) and a battery test channel (BTS-610, Neware, Shenzhen, China) with a voltage window range from 0.01–3.0 V (vs. Li/Li^+^). After each charging or discharging procedure, the batteries are put on hold for 10 s. For the rate test, the number of cycles per current density of the battery was set at 10 times. Electrochemical impedance spectroscopy (EIS) was carried out in the frequency range of 0.01–100,000 Hz with an applied perturbation voltage of 5 mV.

## 4. Conclusions

In summary, we engineered highly amorphous GeO_2_ and carbon composite nanofibers by electrospinning, preoxidation and carbonization. The uniformly distributed GeO_2_ nanoparticles are decorated in the carbon framework, and the high-specific area supplies abundant electrochemical reaction sites for fast lithium diffusion. The amorphous carbon nanofiber not only buffers the induced volume changes but also prevents active material agglomeration. The obtained electrodes exhibit highly reversible alloying and conversion processes during the whole cycling process. The corresponding reversible capacity is 747 mAh g^−1^ after 300 cycles at a current density of 0.3 A g^−1^. In addition, the results of CV testing and calculations indicate that the capacitive-controlled contributions are 21.3%, 26.5%, 30.8%, 34.5% and 37.4% at scanning speeds of 0.2, 0.4, 0.6, 0.8 and 1.0 mV s^−1^, respectively. The facile preparation of an amorphous structure promotes the practical application of GeO_2_ as a next-generation LIB anode material.

## Data Availability

The data presented in this study are available on request from the corresponding author.

## Sample Availability

Samples of the compounds are not available from the authors.

## References

[1] Peng T., Guo W., Zhang Q., Zhang Y., Chen M., Wang Y., Yan H., Lu Y., Luo Y. (2018). Uniform coaxial CNT@Li_2_MnSiO_4_@C as advanced cathode material for lithium-ion battery. Electrochim. Acta.

[2] Bai X., Li D., Zhang D., Yang S., Pei C., Sun B., Ni S. (2023). Boosting high-rate lithium storage in Li_3_VO_4_ via a honeycomb structure design and electrochemical reconstruction. J. Mater. Chem. A.

[3] Li M., Lu J., Chen Z., Amine K. (2018). 30 Years of Lithium-Ion Batteries. Adv. Mater..

[4] Liu C., Fang X., Peng H., Li Y., Yang Y. (2023). Fabrication of Composite Gel Electrolyte and F-Doping Carbon/Silica Anode from Electro-Spun P(VDF-HFP)/Silica Composite Nanofiber Film for Advanced Lithium-Ion Batteries. Molecules.

[5] Xie Y., Cao J., Wang X., Li W., Deng L., Ma S., Zhang H., Guan C., Huang W. (2021). MOF-Derived Bifunctional Co0.85Se Nanoparticles Embedded in N-Doped Carbon Nanosheet Arrays as Efficient Sulfur Hosts for Lithium–Sulfur Batteries. Nano Lett..

[6] Peng T., Liu C., Hou X., Zhang Z., Wang C., Yan H., Lu Y., Liu X., Luo Y. (2017). Control Growth of Mesoporous Nickel Tungstate Nanofiber and Its Application as Anode Material for Lithium-Ion Batteries. Electrochim. Acta.

[7] Wang C., Han Q., Xie R., Wang B., He T., Xie W., Tang Q., Li Y., Xu J., Yu B. (2020). Fabrication of petal-like Ni3S2 nanosheets on 3D carbon nanotube foams as high-performance anode materials for Li-ion batteries. Electrochim. Acta.

[8] Bai Z., Yang Y., Zhang D., Wang Y., Guo Y., Yan H., Chu P.K., Luo Y. (2021). Carbon-encapsulated nanosphere-assembled MoS2 nanosheets with large interlayer distance for flexible lithium-ion batteries. J. Solid State Electrochem..

[9] Sun X., Xie W., Luo F. (2023). Nanoarchitectonics of multilayered NiO submicron flakes for ultrafast and stable lithium storage. J. Alloys Compd..

[10] Cao K., Jia Y., Wang S., Huang K.-J., Liu H. (2021). Mn_3_O_4_ nanoparticles anchored on carbon nanotubes as anode material with enhanced lithium storage. J. Alloys Compd..

[11] Guo Y., Zhang D., Bai Z., Yang Y., Wang Y., Cheng J., Chu P.K., Luo Y. (2022). MXene nanofibers confining MnOx nanoparticles: A flexible anode for high-speed lithium ion storage networks. Dalton Trans..

[12] Sun X., Jing M., Dong H., Xie W., Luo F. (2023). CuO-ZnO submicroflakes with nanolayered Al_2_O_3_ coatings as high performance anode materials in lithium-ion batteries. J. Alloys Compd..

[13] Chen M., Liu F.-M., Zhao H., Chen S.-S., Qian X., Yuan Z.-Y., Wan R. (2022). In situ encapsulation of iron oxide nanoparticles into nitrogen-doped carbon nanotubes as anodic electrode materials of lithium ion batteries. Phys. Chem. Chem. Phys..

[14] Wang K., Ye W., Yin W., Chai W., Tang B., Rui Y. (2019). One-step synthesis of MOF-derived Ga/Ga_2_O_3_@C dodecahedra as an anode material for high-performance lithium-ion batteries. Dalton Trans..

[15] Qu J., Xiao J., Wang T., Legut D., Zhang Q. (2020). High Rate Transfer Mechanism of Lithium Ions in Lithium–Tin and Lithium–Indium Alloys for Lithium Batteries. J. Phys. Chem. C.

[16] Lang W., Yue C., Dang M., Wang G., Chen Y., Hu F., Liu Z., Shu J. (2023). Germanium decorated on three dimensional graphene networks as binder-free anode for Li-ion batteries. J. Power Sources.

[17] Zhang Y., Wang Y., Kong D., Yang Y., Wang Y., Guo Y., Lu Y., Kim J.-K., Luo Y. (2021). In situ growth of Sn nanoparticles confined carbon-based TiO_2_/TiN composite with long-term cycling stability for sodium-ion batteries. Electrochim. Acta.

[18] Shen D., Jia M., Li M., Fu X., Liu Y., Dong W., Yang S. (2023). High Coulomb Efficiency Sn–Co Alloy/rGO Composite Anode Material for Li ion Battery with Long Cycle Life. Molecules.

[19] Liu H., He Y., Cao K., Wang S., Jiang Y., Liu X., Huang K.-J., Jing Q.-S., Jiao L. (2021). Stimulating the Reversibility of Sb2S3 Anode for High-Performance Potassium-Ion Batteries. Small.

[20] Liu X., Wu X.-Y., Chang B., Wang K.-X. (2020). Recent progress on germanium-based anodes for lithium ion batteries: Efficient lithiation strategies and mechanisms. Energy Storage Mater..

[21] Zhao Y., Manthiram A. (2015). High-Capacity, High-Rate Bi–Sb Alloy Anodes for Lithium-Ion and Sodium-Ion Batteries. Chem. Mater..

[22] Wang Q., Xu J., Shen G., Guo Y., Zhao X., Xia Y., Sun H., Hou P., Xie W., Xu X. (2019). Large-scale carbon framework microbelts anchoring ultrafine SnO_2_ nanoparticles with enhanced lithium storage properties. Electrochim. Acta.

[23] Han L., Wei Q., Chen H., Tang J., Wei M. (2021). Open-framework germanates derived GeO_2_/C nanocomposite as a long-life and high-capacity anode for lithium-ion batteries. J. Alloys Compd..

[24] Eisenmann T., Birrozzi A., Mullaliu A., Asenbauer J., Giuli G., Trapananti A., Olivi L., Moon H., Geiger D., Kaiser U. (2021). Impact of Crystal Density on the Electrochemical Behavior of Lithium-Ion Anode Materials: Exemplary Investigation of (Fe-Doped) GeO_2_. J. Phys. Chem. C.

[25] McNulty D., Geaney H., Buckley D., O’Dwyer C. (2018). High capacity binder-free nanocrystalline GeO_2_ inverse opal anodes for Li-ion batteries with long cycle life and stable cell voltage. Nano Energy.

[26] Cao K., Zheng R., Wang S., Shu J., Liu X., Liu H., Huang K.-J., Jing Q.-S., Jiao L. (2020). Boosting Coulombic Efficiency of Conversion-Reaction Anodes for Potassium-Ion Batteries via Confinement Effect. Adv. Funct. Mater..

[27] Wang Y., Zhang D., Yang Y., Guo Y., Bai Z., Chu P.K., Luo Y. (2021). Three-dimensional nano/micro-structured porous MoP/CNTs microspheres as high-capacity anode for lithium-ion batteries. J. Alloys Compd..

[28] Yan S., Song H., Lin S., Wu H., Shi Y., Yao J. (2019). GeO_2_ Encapsulated Ge Nanostructure with Enhanced Lithium-Storage Properties. Adv. Funct. Mater..

[29] Wu J., Luo N., Huang S., Yang W., Wei M. (2019). Reversible conversion reaction of GeO_2_ boosts lithium-ion storage via Fe doping. J. Mater. Chem. A.

[30] Hohn N., Wang X., Giebel M.A., Yin S., Müller D., Hetzenecker A.E., Bießmann L., Kreuzer L.P., Möhl G.E., Yu H. (2020). Mesoporous GeOx/Ge/C as a Highly Reversible Anode Material with High Specific Capacity for Lithium-Ion Batteries. ACS Appl. Mater. Interfaces.

[31] Wu J., Tang A., Wang K., Huang S., Wei M. (2022). Self-Optimizing Effect in Lithium Storage of GeO_2_ Induced by Heterointerface Regulation. Small.

[32] Medvedev A.G., Mikhaylov A.A., Grishanov D.A., Yu D.Y.W., Gun J., Sladkevich S., Lev O., Prikhodchenko P.V. (2017). GeO_2_ Thin Film Deposition on Graphene Oxide by the Hydrogen Peroxide Route: Evaluation for Lithium-Ion Battery Anode. ACS Appl. Mater. Interfaces.

[33] Guo Y., Zhang Y., Wang Y., Zhang D., Lu Y., Luo R., Wang Y., Liu X., Kim J.-K., Luo Y. (2019). Vertically aligned ultrathin MoS_2_ nanosheets grown on graphene-wrapped hollow carbon microtubes derived from loofah sponge as advanced anodes for highly reversible lithium storage. Electrochim. Acta.

[34] Zhang W., Pang H., Sun W., Lv L.-P., Wang Y. (2017). Metal-organic frameworks derived germanium oxide nanosheets for large reversible Li-ion storage. Electrochem. Commun..

[35] Liu X., Zhang K., Wang Q., Cui D., Gao G., Wang C., Hu J., Yao Y., Li Y. (2023). Three-dimensional honeycomb-like porous carbon embedded with Ge nanoparticles anode composites for ultrastable lithium storage. J. Alloys Compd..

[36] Liu F., Wang Y., Shi J., Lin J., Zhou W., Pan A. (2019). A new strategy to prepare Ge/GeO_2_-reduced graphene oxide microcubes for high-performance lithium-ion batteries. Electrochim. Acta.

[37] Gavrilin I.M., Kudryashova Y.O., Kuz’mina A.A., Kulova T.L., Skundin A.M., Emets V.V., Volkov R.L., Dronov A.A., Borgardt N.I., Gavrilov S.A. (2021). High-rate and low-temperature performance of germanium nanowires anode for lithium-ion batteries. J. Electroanal. Chem..

[38] Koo J.-H., Paek S.-M. (2021). Microwave-Assisted Synthesis of Ge/GeO2-Reduced Graphene Oxide Nanocomposite with Enhanced Discharge Capacity for Lithium-Ion Batteries. Nanomaterials.

[39] Choi S.H., Jung K.Y., Kang Y.C. (2015). Amorphous GeOx-Coated Reduced Graphene Oxide Balls with Sandwich Structure for Long-Life Lithium-Ion Batteries. ACS Appl. Mater. Interfaces.

[40] Li X., Liang J., Hou Z., Zhu Y., Wang Y., Qian Y. (2014). Coordination complex pyrolyzation for the synthesis of nanostructured GeO_2_ with high lithium storage properties. Chem. Commun..

[41] Wang Z., Zhang X., Yan Y., Zhang Y., Wang Y., Qin C., Bakenov Z. (2019). Nanoporous GeO_2_/Cu/Cu_2_O network synthesized by dealloying method for stable Li-ion storage. Electrochim. Acta.

[42] Yan Y., Liu Y., Zhang Y., Qin C., Yu H., Bakenov Z., Wang Z. (2021). Sn modified nanoporous Ge for improved lithium storage performance. J. Colloid Interface Sci..

[43] Deng L., Li W., Li H., Cai W., Wang J., Zhang H., Jia H., Wang X., Cheng S. (2020). A Hierarchical Copper Oxide–Germanium Hybrid Film for High Areal Capacity Lithium Ion Batteries. Front. Chem..

[44] Wei X., Li W., Zeng L., Yu Y. (2016). Influence of Carbon Matrix Dimensions on the Electrochemical Performance of Germanium Oxide in Lithium-Ion Batteries. Part. Part. Syst. Charact..

[45] He X., Hu Y., Shen Z., Chen R., Wu K., Cheng Z., Zhang X.W., Pan P. (2017). Channelized carbon nanofiber with uniform-dispersed GeO_2_ as anode for long-lifespan lithium-ion batteries. J. Alloys Compd..

[46] Sun X., Luo F. (2023). Sodium Storage Properties of Carbonaceous Flowers. Molecules.

[47] Yang Q., Sun T., Yu J.-Y., Ma J.-X. (2016). Electrospinning of GeO_2_–C fibers and electrochemical application in lithium-ion batteries. Chin. Chem. Lett..

[48] Han L., Tang J., Yang R., Wei Q., Wei M. (2021). Stable Li-ion storage in Ge/N-doped carbon microsphere anodes. Nanoscale.

[49] Lin Y., Zhong K., Zheng J., Liang M., Xu G., Feng Q., Li J., Huang Z. (2021). Fluorine-Doped GeO_2_@C Composite with Abundant Oxygen Vacancies for High-Capacity Lithium-Ion Batteries. ACS Appl. Energy Mater..

[50] Youn D.H., Patterson N.A., Park H., Heller A., Mullins C.B. (2016). Facile Synthesis of Ge/N-Doped Carbon Spheres with Varying Nitrogen Content for Lithium Ion Battery Anodes. ACS Appl. Mater. Interfaces.

[51] Xie Y., Ao J., Zhang L., Shao Y., Zhang H., Cheng S., Wang X. (2023). Multi-functional bilayer carbon structures with micrometer-level physical encapsulation as a flexible cathode host for high-performance lithium-sulfur batteries. Chem. Eng. J..

